# Severe acute respiratory syndrome coronavirus 2 seroprevalence among personnel in the healthcare facilities of Croatia, 2020

**DOI:** 10.1590/0037-8682-0458-2020

**Published:** 2020-08-26

**Authors:** Tatjana Vilibic-Cavlek, Vladimir Stevanovic, Irena Tabain, Ljiljana Betica-Radic, Dario Sabadi, Ljiljana Peric, Maja Bogdanic, Maja Vilibic, Branko Kolaric, Boris Kudumija, Goranka Petrovic, Anna Mrzljak, Svjetlana Karabuva, Irena Hrstic, Krunoslav Capak, Jasmina Kucinar, Vladimir Savic, Ljubo Barbic

**Affiliations:** 1 Croatian Institute of Public Health, Department of Virology, Zagreb, Croatia.; 2 University of Zagreb, School of Medicine, Zagreb, Croatia.; 3 University of Zagreb, Faculty of Veterinary Medicine, Department of Microbiology and Infectious Diseases with Clinic, Zagreb, Croatia.; 4 General Hospital Dubrovnik, Department of Infectious Diseases, Dubrovnik, Croatia.; 5 Clinical Hospital Center Osijek, Department of Infectious Diseases, Osijek, Croatia.; 6 Josip Juraj Strossmayer University of Osijek, Medical Faculty, Osijek, Croatia.; 7 Sestre Milosrdnice University Hospital Centre, Department of Psychiatry, Zagreb, Croatia.; 8 Andrija Stampar Teaching Institute of Public Health, Department of Public Health Gerontology, Zagreb, Croatia.; 9 University of Rijeka, Faculty of Medicine, Rijeka, Croatia.; 10 B. Braun Avitum Polyclinic for Internal Medicine and Dialysis, Zagreb, Croatia.; 11 Croatian Institute of Public Health, Department of Epidemiology, Zagreb, Croatia.; 12 Merkur University Hospital, Department of Medicine, Zagreb, Croatia.; 13 University Hospital Center Split, Department of Infectious Diseases, Split, Croatia.; 14 General Hospital Pula, Department of Medicine, Pula, Croatia.; 15 Croatian Institute of Public Health, Environmental Health Department, Zagreb, Croatia.; 16 Istria County Institute of Public Health, Department of Serology and Immunology, Pula, Croatia.; 17 Croatian Veterinary Institute, Laboratory for Virology and Serology, Zagreb, Croatia.

Dear Editor,

Coronavirus disease (COVID-19) is an acute respiratory disease caused by severe acute respiratory syndrome coronavirus 2 (SARS-CoV-2). After the emergence of COVID-19 in Wuhan (China) in December 2019, the number of global cases increased significantly, and in March 2020, the World Health Organization (WHO) declared it a pandemic^1^. Human-to-human transmission of SARS-CoV-2 occurs primarily through close contact and respiratory droplets. The clinical spectrum of COVID-19 varies from asymptomatic infection to severe and even fatal pneumonia^2^. Since the initial surveillance focused primarily on symptomatic COVID-19 patients, the full spectrum of the disease or extent of mild or asymptomatic infection not requiring medical attention was unclear. Statistical estimates of COVID-19 incidence indicated that the number of asymptomatic cases was significant^3^. Healthcare personnel, particularly infectious disease and intensive care physicians, emergency doctors, epidemiologists, microbiologists, nurses, and even cleaning staff, have frequent direct or indirect exposure to patients or infected materials. Estimation of asymptomatic and mild cases, including healthcare workers (HCWs), is important for understanding COVID-19 transmission, providing insight into the epidemic spread^4^.

In Croatia, the first COVID-19 case was confirmed on February 25, 2020. A total of 3,272 cases and 113 deaths from COVID-19 were reported by July 7, 2020 (data of the Croatian Institute of Public Health). To date, there are no data on the prevalence of SARS-CoV-2 infection in healthcare settings. We analyzed the seroprevalence of COVID-19 in different professionally exposed populations.

From April 25 to May 24, 2020, when the COVID-19 epidemic curve was approaching the end of the first wave in Croatia, a total of 592 serum samples from HCWs and allied/auxiliary HCWs were tested for the presence of SARS-CoV-2 antibodies. Convenient samples were collected from six counties with a high incidence of COVID-19. WHO defined HCWs as all people at healthcare facilities involved in the provision of care for COVID-19 patients as well as those who may not have provided direct care to patients but may have come in contact with patients' body fluids, potentially contaminated materials or devices, and equipment linked to patients or environmental surfaces^3^. The study group included: a) healthcare professionals working in different hospital/emergency wards; b) laboratory personnel included in the phlebotomy and SARS-CoV-2 diagnostic units; c) patient transporters; d) cleaning personnel; and e) others (social workers, physical therapists, and administrative workers). All participants included in the study filled out a questionnaire regarding their demographic information, clinical symptoms, and possible exposure to COVID-19.

Serum samples were initially screened for the presence of SARS-CoV-2 IgG antibodies. Reactive samples were further tested for IgM/IgA antibodies. Serological tests were performed with a commercial enzyme-linked immunosorbent assay (ELISA) using spike glycoprotein (S) and nucleocapsid protein (N) antigens (Vircell, Granada, Spain). All positive samples were confirmed using a virus neutralization test (VNT). For VNT, the SARS-CoV-2 HR1/8933 was isolated in Vero E6 cells from the nasopharyngeal swab of a COVID-19 patient. Maximum cytopathic effect was visible on the 4^th^ day, and virus replication was confirmed by reverse-transcriptase polymerase chain reaction (RT-PCR). Heat-inactivated serum samples (56°C/30 min) were tested in duplicate in 96-well plates. Two-fold serum dilutions starting from 1:2 were prepared and mixed with the equal-volume (25 µL) suspension containing median tissue culture infectious dose (100 TCID_50_) of the virus. After 1 h of incubation (37°C) in a CO_2_ incubator, a 50-µL suspension of Vero E6 cells (concentration: 2×10^5^ cells/mL) was added to each well and incubated for 4 days. The antibody titer was defined as the reciprocal value of the highest serum dilution that showed 100% neutralization in at least half of the infected wells. Titers >8 were considered positive. 

The study protocol was approved by the Ethics Committee of the Croatian Institute of Public Health. Written informed consent was obtained from all participants.

Table 1 shows the characteristics of study participants and potential risk exposures to COVID-19. The tested group included 152 (25.7%) men and 440 (74.3%) women, aged 20 to 65 years. As a possible risk factor, 116 (19.6%), 108 (18.2%), and 62 (10.5%) participants reported contact with a confirmed COVID-19 patient, participation in large community events, and travelling to areas with documented COVID-19 transmission, respectively. Clinical symptoms consistent with COVID-19 were reported by 300 (50.7%) participants. SARS-CoV-2 RT-PCR was performed for 180 (30.4%) participants.

**TABLE 1:** Epidemiological data and occupational exposure of study participants.

Characteristic	N (%) tested
Sex	
Male	152 (25.7%)
Female	440 (74.3%)
Travelling to an area with documented COVID-19 circulation	62 (10.5%)
Contact with a confirmed COVID-19 patient	116 (19.6%)
Participation in large community events	108 (18.2%)
Occupational exposure	
Infectious disease ward staff	150 (25.3%)
Internal medicine ward staff	24 (4.0%)
Anesthesiology ward staff	12 (2.0%)
Surgery ward staff	13 (2.2%)
Gynecology ward staff	23 (3.9%)
Psychiatry ward staff	31 (5.2%)
Pediatric ward staff	26 (4.4%)
Physiotherapy staff	18 (3.0%)
Emergency medicine staff	23 (3.9%)
Radiology staff	11 (1.9%)
Hemodialysis unit staff	34 (5.7%)
Microbiology (COVID-19 diagnostics) staff	29 (4.9%)
Epidemiology/public health staff	21 (3.5%)
Laboratory staff	10 (1.7%)
Patient transporters	33 (5.6%)
Cleaning personnel	15 (2.6%)
Dental medicine staff	15 (2.6%)
General practice staff	48 (8.1%)
Nursing homes/eldercare facility staff	22 (3.8%)
Others (social workers, administrative workers)	34 (5.7%)
Clinical symptoms consistent with COVID-19	300 (50.7%)
Fever ≥38°C	68 (11.5%)
Chills	62 (10.5%)
Fatigue	152 (25.7%)
Muscle aches (myalgia)	110 (18.6%)
Sore throat	143 (24.1%)
Cough	144 (24.3%)
Runny nose (rhinorrhea)	193 (32.6%)
Shortness of breath	41 (6.9%)
RT-PCR testing positive for SARS-CoV-2	180 (30.4%)
Influenza vaccination in 2019/2020	128 (21.6%)

**COVID-19:** coronavirus disease; **RT-PCR:** reverse-transcriptase polymerase chain reaction; **SARS-CoV-2:** severe acute respiratory syndrome coronavirus 2.

Using ELISA, IgG and IgM/IgA antibodies against SARS-CoV-2 were detected in 16 (2.7%) and 9 (1.5%) participants, respectively. Neutralizing antibodies were confirmed in 9 (1.5%) participants (titers: 32 to 256). Five seropositive HCWs were also RT-PCR-positive. All of them showed both IgG (antibody index [AI]: 17.08-37.31) and IgM/IgA (AI: 20.70-49.60) antibodies with neutralizing antibody titers from 32 to 256. Seven seropositive persons were healthcare professionals (medical doctor, nurses, and technicians), while two were administrative workers. All but one HCW worked in the infectious disease department. Three of them reported experiencing clinical symptoms in the past 2 months, while six were asymptomatic. 

As of April 8, 2020, more than 22,000 cases of COVID-19 among HCWs from 52 countries have been reported to WHO^1^. Using RT-PCR, the COVID-19 prevalence of 6% was found in HCWs at two Dutch teaching hospitals (Breda and Tilburg). There was no clustering of infected HCWs in any specific department^5^. Two studies from the United Kingdom showed that 18% of symptomatic HCWs^6^and 3% of asymptomatic HCWs tested RT-PCR-positive for SARS-CoV-2^7^.

Data are limited on the seroprevalence of COVID-19 among HCWs. In this study, using ELISA, SARS-CoV-2 IgG antibodies were detected in 2.7% of participants, while neutralizing antibodies were detected in 1.5% of participants, indicating a low seroprevalence among HCWs in Croatia. Preliminary results from the Croatian Institute of Public Health showed a slightly higher seroprevalence rate in the general population (2.3%). While a positive ELISA result, even if specific, provides evidence of prior infection with SARS-CoV-2, it does not ensure protective immunity, since neutralizing antibodies correlate with protection. Screening with SARS-CoV-2 IgG ELISA (high sensitivity) followed by VNT (high specificity) is a reliable approach for seroepidemiological studies^8^.

A seroprevalence study from Germany analyzed three groups of HCWs: a high-risk group with daily contact to known/suspected COVID-19 patients, an intermediate-risk group with daily contact to patients without known/suspected COVID-19 infection at admission, and a low-risk group without patient contact. As in this study, the overall seroprevalence of SARS-CoV-2 was low (1.6%). The seropositivity was higher in the intermediate-risk group compared to the high-risk group (5.4% vs. 1.2%); however, this difference was not statistically significant. Four of the five seropositive subjects reported experiencing COVID-19-associated symptoms in the past 3 months^9^. Another study conducted in Barcelona found that 9.3% of 578 tested HCWs were seropositive to SARS-CoV-2^10^. In the present study, three seropositive HCWs reported experiencing COVID-19-consistent clinical symptoms, while six were asymptomatic.

In conclusion, the SARS-CoV-2 seroprevalence in healthcare facilities in Croatia is low, indicating that protective measures have been effective. However, further large-scale seroepidemiological studies are required to confirm this observation.
